# Endophytic bacterial communities in ungerminated and germinated seeds of commercial vegetables

**DOI:** 10.1038/s41598-023-47099-4

**Published:** 2023-11-14

**Authors:** Jacquelinne J. Acuña, Jingming Hu, Nitza G. Inostroza, Tamara Valenzuela, Pablo Perez, Slava Epstein, Angela Sessitsch, Qian Zhang, Milko A. Jorquera

**Affiliations:** 1 Laboratorio de Ecología Microbiana Aplicada (EMALAB), Departamento de Ciencias Químicas y Recursos Naturales, Universidad de La Frontera, Ave. Francisco Salazar, 01145 Temuco, Chile; 2 Center of Plant, Soil Interaction and Natural Resources Biotechnology, Scientific and Technological Bioresource Nucleus (BIOREN), Universidad de La Frontera, Ave. Francisco Salazar, 01145 Temuco, Chile; 3 Millennium Institute Center for Genome Regulation (MI-CGR), Valenzuela Puelma 10207, 7800003 Santiago, La Reina Chile; 4 Fujian Provincial Key Laboratory for Coastal Ecology and Environmental Studies, Xiamen University, Xiamen, 361102 China; 5 College of the Environment and Ecology, Xiamen University, Xiamen, 361102 China; 6 Programa de Doctorado en Ciencias de Recursos Naturales, Universidad de La Frontera, Ave. Francisco Salazar 01145, Temuco, Chile; 7 College of Science, Northeastern University, 360 Huntington Ave., Boston, MA 02115 USA; 8 Health & Bioresources, AIT Austrian Institute of Technology, Konrad-Lorenz-Straße 24, 3430 Tulln, Austria

**Keywords:** Applied microbiology, Microbial communities, Plant biotechnology

## Abstract

Chile is a prominent seed exporter globally, but the seed microbiome of vegetables (46% of seeds) and its role in the early stages of plant growth have remained largely unexplored. Here, we employed DNA metabarcoding analysis to investigate the composition and putative functions of endophytic bacterial communities in ungerminated and germinated seeds of the commercial vegetables *Apiaceae* (parsley and carrot), *Asteraceae* (lettuce), *Brassicaceae* (cabbage and broccoli), and *Solanaceae* (tomato). Bacterial quantification showed 10^4^ to 10^8^ copies of the 16S rRNA gene per gram of ungerminated and germinated seeds. Alpha diversity analysis (e.g., Chao1, Shannon, and Simpson indices) did not indicate significant differences (Kruskal–Wallis test) between ungerminated and germinated seeds, except for *Solanaceae*. However, beta diversity (PCoA) analysis showed distinctions (Adonis test) between ungerminated and germinated seeds, except *Apiaceae*. Pseudomonadota and Bacillota were identified as the dominant and specialist taxa in both ungerminated and germinated seed samples. Chemoheterotrophy and fermentation were predicted as the main microbial functional groups in the endophytic bacterial community. Notably, a considerable number of the 143 isolated endophytic strains displayed plant growth-promoting traits (10 to 64%) and biocontrol activity (74% to 82%) against plant pathogens (*Xanthomonas* and *Pseudomonas*). This study revealed the high variability in the abundance, diversity, composition, and functionality of endophytic bacteria between ungerminated and germinated seeds in globally commercialized vegetables. Furthermore, potential beneficial endophytic bacteria contained in their seed microbiomes that may contribute to the microbiome of the early stages, development, growth and progeny of vegetables were found.

## Introduction

Agriculture is recognized as a sector that is highly relevant for Chile’s economy and development, not only as a source of employment and its contribution to Chile’s gross domestic product (projected to ~ 7% for 2030) but also as an exporter opening diverse markets for Chilean agricultural products around the globe^1^. In this context, Chile is recognized as one of the prominent seed exporters in the Southern Hemisphere and ranks among the top-10 global seed producers according to the data published by The International Seed Federation (ISF) and Chile’s National Association of Seed Producers (ANPROS)^2,3^. Thus, Chile has emerged as a key player in the production and innovation of seeds worldwide^4^, employing 68,000 people and acting as a source of off-season seeds^5^. Based on ISF’s data analyzed by ANPROS during 2018^2,3^, the total seed exports grew from USD$ 338 MM in 2017 to USD$ 392 MM in 2018, which was a 16% increase in value and an 18% increase in amount; vegetables represent the highest amount of exported seeds, with a percentage of 52.9% (USD$ 197,046,431), followed by maize with 23.2% (USD$ 86,586,326), and sunflower with 8.9% (USD$ 33,297,824). Within vegetables, the main exported seeds in 2018 belonged to broccoli (USD$27,348,294; 133 metric tons), watermelon (USD$26,099,438; 26 metric tons), pepper (USD$19,955,873; 27 metric tons), cabbage (USD$17,858,084; 272 metric tons) and cauliflower (USD$17,005,685; 113 metric tons).

During the last decade, numerous studies have revealed that plant microbiomes play a pivotal role in the development, growth, and resilience of various plants, including commercially relevant species^6^. Seeds are not an exception, harboring complex microbial communities that may also exert beneficial or deleterious effects on plant growth and health^7^. Studies have revealed that seeds can host members of Pseudomonadota (*Pseudomonas* and *Pantoea*), Bacillota (*Bacillus*), Actinomycetota (*Streptomyces*), and Bacteroidota (*Bacteroides*)^8,9^ and are often specific and distinct from the microbiota of other plant compartments^10^. This is in line with studies showing that the abundance, composition, and activity of bacterial communities vary by plant compartment^11^. Furthermore, diverse biotic (e.g., plant phenological stages, plant genotype, plant exudates and pathogen attack) and abiotic (e.g., climate, edaphic properties, fertilization practices) parameters influence the establishment of plant microbiota^12–15^. Overall, seed microbiota often possess interesting plant growth-promoting (PGP) functions and seem to be particularly important for early seed vigor^16^. The composition of microbial communities in seeds has traditionally been explored through culture–based analysis; however, culture-independent molecular techniques are less biased and appropriate to assess the diversity of seed microbiota.

Notably, seeds harbor adapted endophytic bacteria that can be vertically transferred to progeny plants, contributing to the colonization and establishment of initial microbiomes in the spermosphere and their subsequent establishment of microbial communities in different plant niches^17–19^. During seed germination and plant ontogeny, coordinated biochemical and molecular processes are triggered, particularly under favorable environmental stimuli (light, temperature, water, and soil nutrients)^20^. However, how the structure and activity of endophytic bacterial communities of commercial vegetables respond and adapt to changes during germination and the early stages of seedling growth is still unknown and remains poorly investigated, even under controlled conditions. Several studies have also shown that seeds can harbor beneficial endophytic bacteria (e.g., *Paraburkholderia phytofirmans*), which can be transferred to progenies, providing a new and exciting opportunity for seed biotechnology^9,19^. Therefore, it has been proposed that seed microbiome engineering can be utilized to promote the growth, fitness, and productivity of plants by incorporating desirable specific beneficial microorganisms^21^. Thus, the incorporation of beneficial plant microorganisms, such as those showing PGP and biocontrol activities, in the microbiomes of commercialized seeds might not only contribute to better plant performance but also be useful for the adoption of a system for international phytosanitary certification of seeds^22^. Plant beneficial microorganisms may improve the development, growth and stress tolerance of plants, particularly in regions affected by adverse climatic events (e.g., droughts, heat waves, flood events, etc.) and emerging pathogen attacks because of climate change. Because seed germination represents one of the most crucial stages of plant development and performance and seed microbiota play a crucial role, the exploration of microbiota associated with commercial seeds is important.

Under this scenario, we hypothesized that significant changes in the assembly and functioning of endophytic bacterial communities occur during the germination of commercial vegetable seeds. Thus, the main objective of this study was to investigate the abundance, structure, and putative functionality of endophytic bacteria present in ungerminated and germinated seeds of commercially important vegetables by DNA metabarcoding analysis. In addition, PGP traits were investigated in isolated endophytic bacteria from ungerminated and germinated seeds. Our results provide insights into seed microbiome dynamics during germination and their potential implications for the development and health of commercial vegetables.

## Results

### Abundance of endophytic bacteria

**Figure 1:**
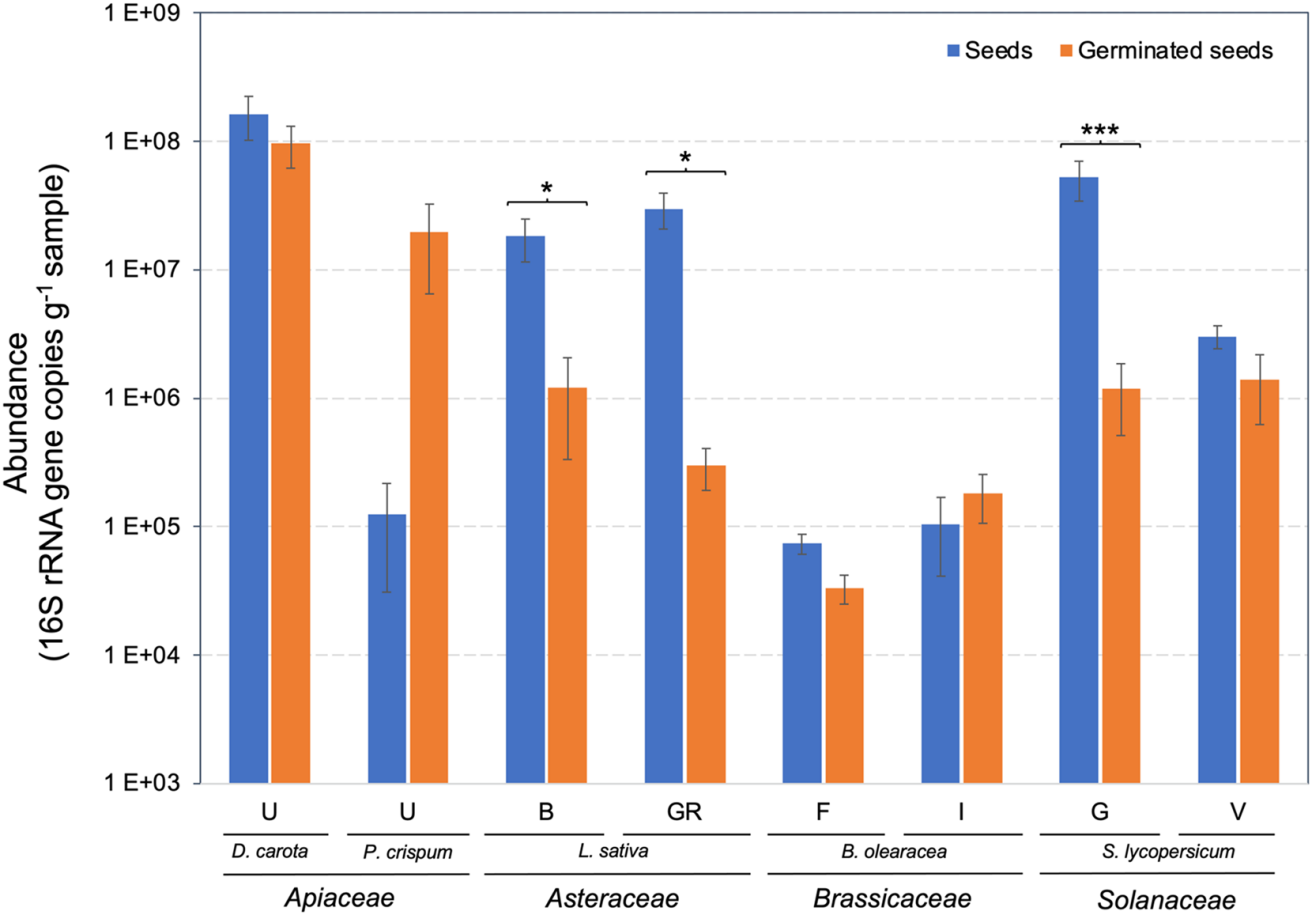
Abundance of endophytic bacteria (16S rRNA gene copies g^–1^ sample) in ungerminated and germinated seeds of the vegetable families *Apiaceae* (parsley *Petroselinum crispum* and carrot *Daucus carota*, undefined varieties [U]), *Asteraceae* (lettuce, *Lactuca sativa*, ‘Brandelier’ [B] and ‘Grand rapids’ [GR] varieties), *Brassicaceae* (cauliflower and broccoli, *Brassica oleracea*, ‘Fausto’ [F] and ‘Italica’ [I] varieties, respectively), and *Solanaceae* (tomato, *Solanum lycopersicum*, ‘Gladiador’ [G] and ‘Velocity’ [V] varieties). Asterisks denote significant differences (*p < 0.05 and ***p < 0.001, Kruskal–Wallis test).

In seeds, the results of qPCR analysis ranged from 1.6 × 10^8^ to 7.4 × 10^4^ 16S rRNA gene copies g^–1^ in *Daucus carota* (undefined [U] variety) and *Brassica oleracea* (‘Fausto’ [F] variety), respectively (Fig. 1). Coincidently, germinated seeds showed higher (9.6 × 10^7^ rRNA gene copies g^–1^) and lower (3.3 × 10^4^ 16S rRNA gene copies g^–1^) numbers in *D. carota* and *B. oleracea* (F variety). Significant differences (Kruskal–Wallis test) between ungerminated and germinated seeds were also observed, such as higher 16S rRNA gene copy numbers in germinated seeds of *Petroselinum crispum* (1.7 × 10^7^ 16S rRNA gene copies g^–1^) than in ungerminated seeds (1.2 × 10^5^ 16S rRNA gene copies g^–1^). In contrast, higher numbers of 16S rRNA gene copies g^–1^ sample were found in ungerminated seeds of *L. sativa* (1.8 × 10^7^ and 2.9 × 10^7^ in ‘Brandelier’ [B] and ‘Grand Rapids’ [GR] varieties, respectively) and *Solanum lycopersicum* (5.2 × 10^7^) than in germinated seeds (1.2 × 10^6^, 2.9 × 10^5^ and 1.2 × 10^6^, respectively).

### Diversity of endophytic bacterial community

**Figure 2:**
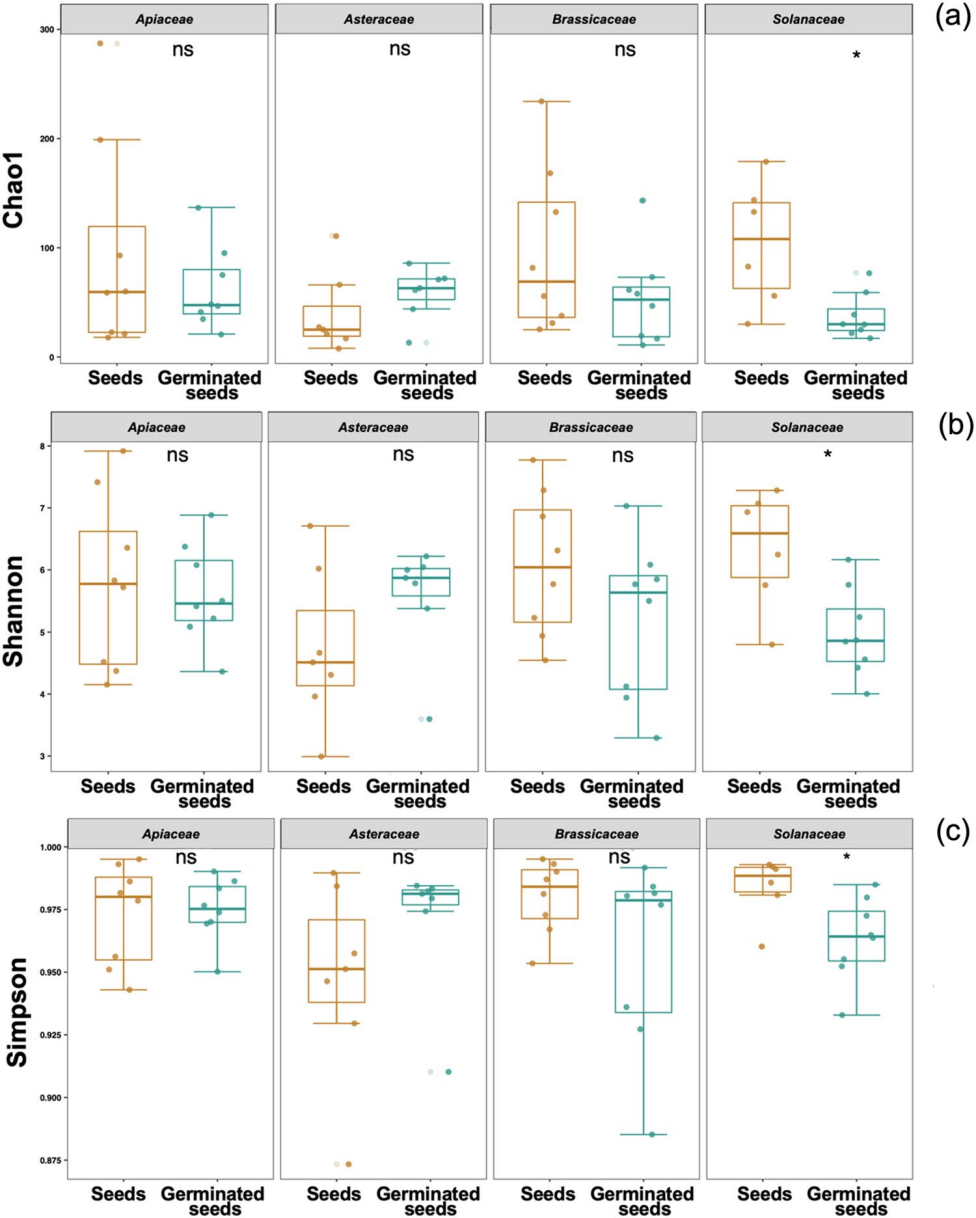
Alpha diversity of endophytic bacterial communities in ungerminated seeds (brown) and germinated seeds (green) of the vegetable families *Apiaceae*, *Asteraceae*, *Brassicaceae*, and *Solanaceae* determined by Chao1 (**a**), Shannon (**b**) and Simpson (**c**) indices. Asterisks denote significant differences (*p < 0.05 and **p < 0.01, Kruskal‒Wallis test). *ns* not significant difference.

Alpha diversity analysis of the sequencing data, represented by Chao1 (number of species), Shannon (relative abundance of each species) and Simpson (dominance of species) indices, did not reveal significant differences (Kruskal–Wallis test) in endophytic bacterial communities between ungerminated and germinated seeds in all vegetables studied, except in *S. lycopersicum* (Fig. 2). The diversity (*p* < 0.01) and dominance of endophytic bacteria in *Solanaceae* significantly decreased after germination, as revealed by the Kruskal–Wallis test. In general, the values of the Chao1, Shannon and Simpson indices varied from 3 to 441, 0.16 to 7.11 and 0.03 to 0.99, respectively.

**Figure 3:**
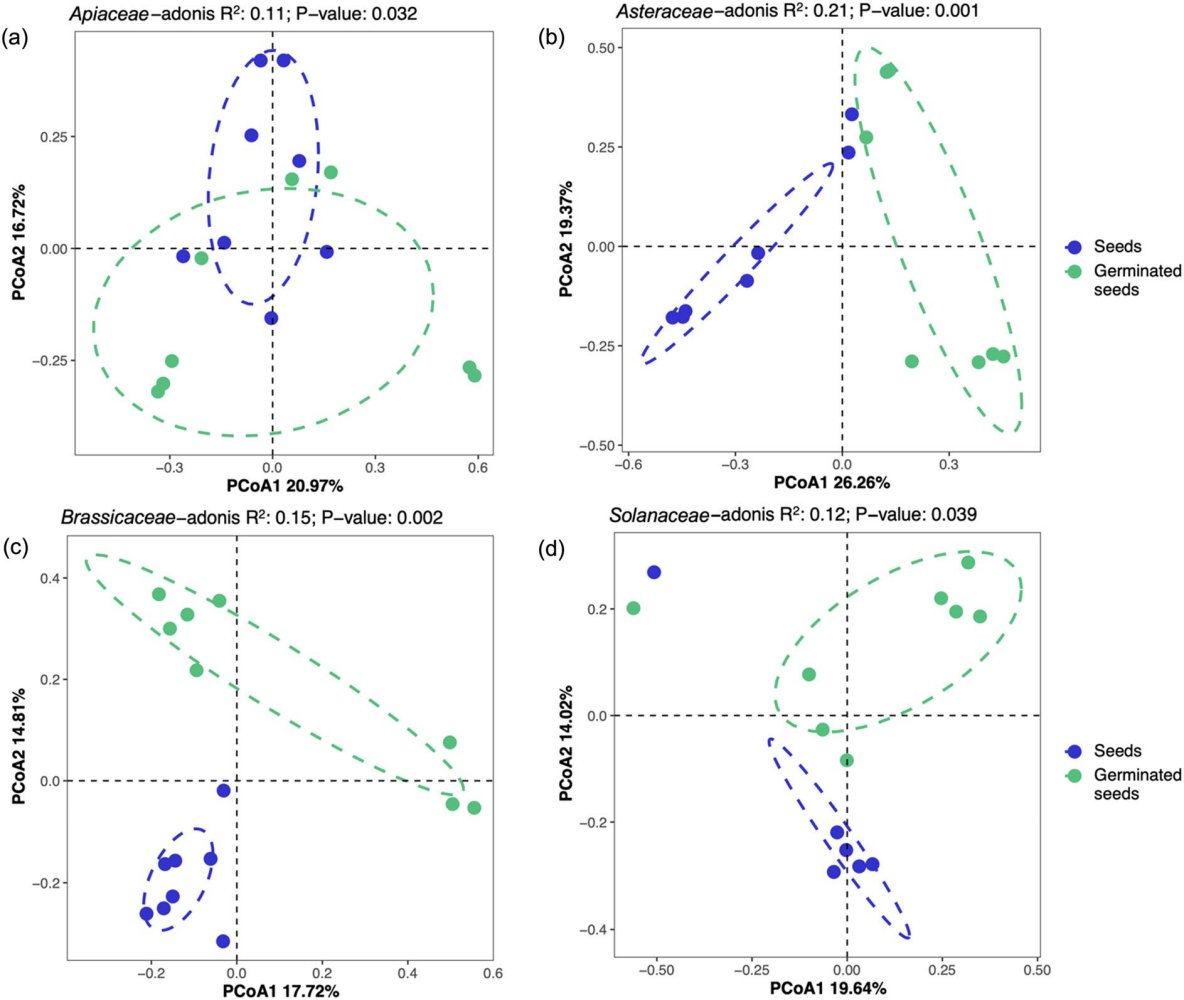
Principal coordinate analysis (PCoA) of endophytic bacterial communities in ungerminated seeds (blue dots) and germinated seeds (green dots) of the vegetable families *Apiaceae* (**a**), *Asteraceae* (**b**), *Brassicaceae* (**c**), and *Solanaceae* (**d**). Differences were analyzed by adonis (*p* < 0.05).

In contrast to alpha diversity, beta diversity analysis revealed significant differences (Adonis test) between ungerminated and germinated seeds, and principal coordinate analysis (PCoA) showed different bacterial communities in ungerminated and germinated seeds of *Asteraceae*, *Brassicaceae*, and *Solanaceae*, whereas the bacterial community of *Apiaceae* seeds showed an overlap between ungerminated and germinated seeds (Fig. 3).

### Taxonomic composition of the endophytic bacterial community

**Figure 4:**
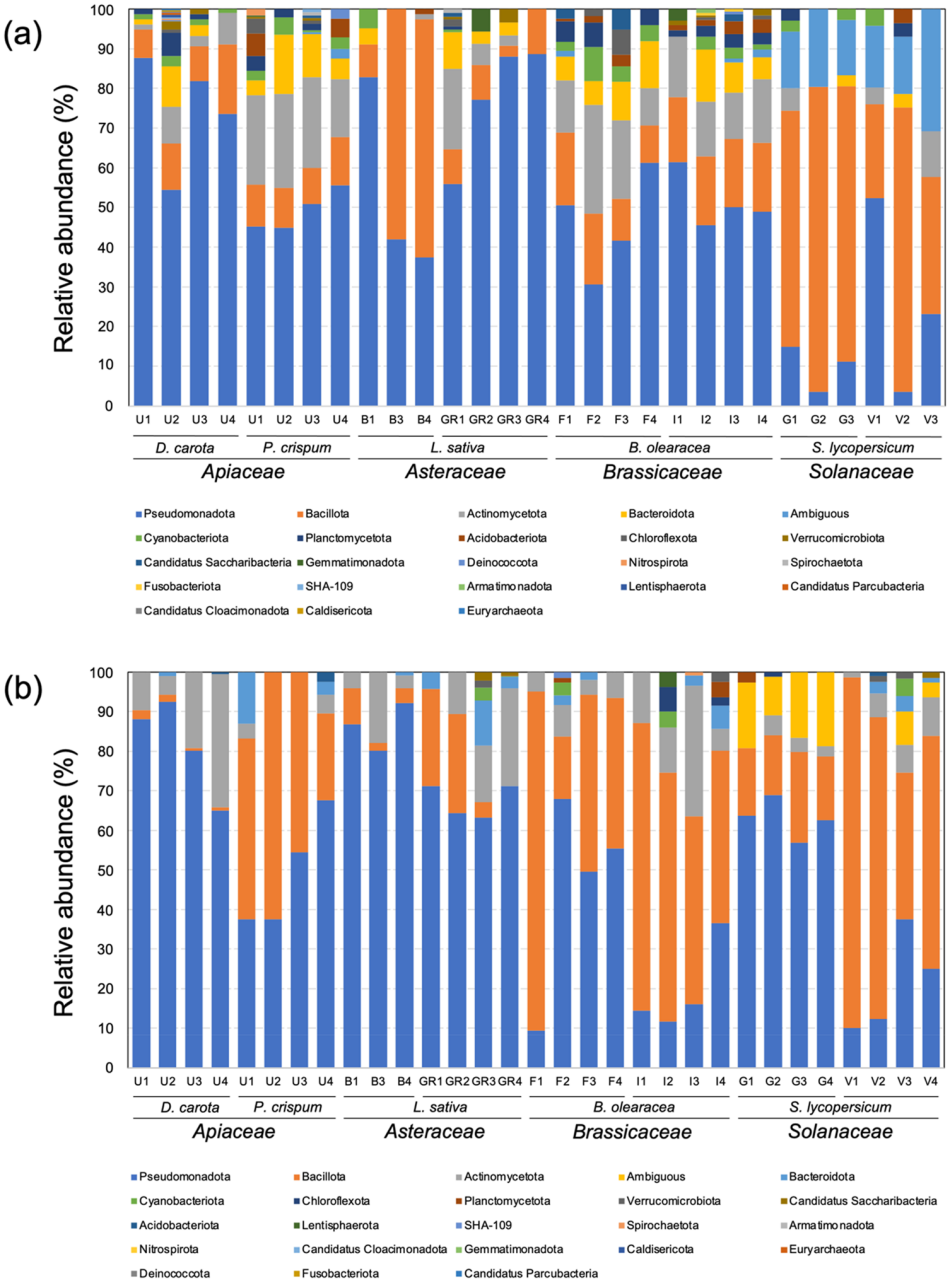
Relative abundances (%) of taxa at the phylum level of endophytic bacterial communities in ungerminated seeds (**a**) and germinated seeds (**b**) of the vegetable families *Apiaceae* (parsley *Petroselinum crispum* and carrot *Daucus carota*, undefined varieties [U]), *Asteraceae* (lettuce, *Lactuca sativa*, ‘Brandelier’ [B] and ‘Grand rapids’ [GR] varieties), *Brassicaceae* (cauliflower and broccoli, *Brassica oleracea*, ‘Fausto’ [F] and ‘Italica’ [I] varieties, respectively), and *Solanaceae* (tomato, *Solanum lycopersicum*, ‘Gladiador’ [G] and ‘Velocity’ [V] varieties).

Regarding the taxonomic composition of the endophytic bacterial community, most plant varieties showed Pseudomonadota as the dominant phylum in ungerminated seeds, ranging from 31.7% (*B. oleracea*, F variety) to 89% (*L. sativa*, G variety), followed by the phyla Actinomycetota (24 to 3%) and Bacillota (18 to 3%) (Fig. 4a). Similarly, samples of *L. sativa* (B variety) were mainly dominated by Bacillota (58 to 60%) and Pseudomonadota (37 to 83%). In contrast, both varieties of *S. lycopersicum* were mainly dominated by Bacillota (24 to 77%). Other phyla found in ungerminated seeds, but in lower relative abundance, were Bacteroidota, Cyanobacteriota and Planctomycetota. In germinated seeds, the samples of *D. carota*, *L. sativa* (B variety), and *S. lycopersicum* (‘Gladiador’ [G] variety) were mainly dominated by Pseudomonadota (65 to 93%, 80 to 92% and 23 to 67%, respectively), while the samples of *B. oleracea* (‘Italica’ [I] variety) and *S. lycopersicum* (‘Velocity’ [V] variety) were mainly dominated by Bacillota (58 to 60% and 37 to 89%, respectively) (Fig. 4b). Actinomycetota was also observed as a dominant taxon in germinated seeds of *D. carota* and *B. oleracea* (I variety), with relative abundance values ranging from 5 to 34% and 6 to 33%, respectively.

### Specificity–occupancy (SPEC–OCCU) plotting

**Figure 5:**
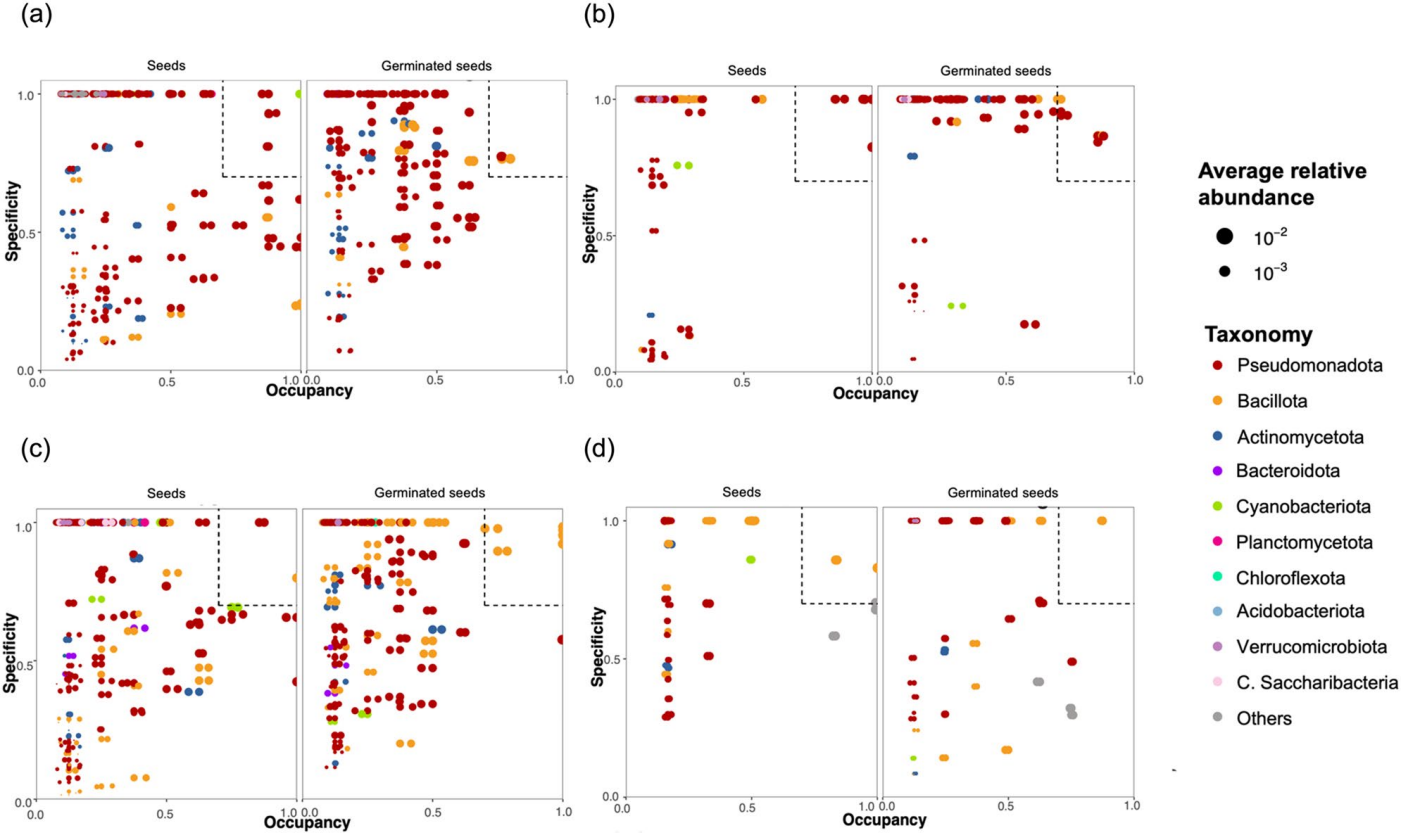
Specialist taxa in the endophytic bacterial community visualized by specificity–occupancy (SPEC–OCCU) plotting in ungerminated and germinated seeds of the vegetable families *Apiaceae* (**a**), *Asteraceae* (**b**), *Brassicaceae* (**c**), and *Solanaceae* (**d**). The *x*-axis represents the occupancy, which is the distribution of OTUs across all samples of ungerminated or germinated seeds, while the *y*-axis represents the specificity (i.e., whether OTUs are also present in another group of seeds or germinated seeds).

To identify the specialist taxa for each vegetable family, OTUs with specificity and occupancy greater than or equal to 0.7 were selected. The SPEC–OCCU plot (Fig. 5) revealed that the specialized varieties at different stages of different vegetables were generally similar but slightly different. In terms of specificity and occupancy, *Asteraceace*, *Solanaceae* and *Brassicaceae* did not differ between ungerminated and germinated seeds.

In addition, the endophytic bacterial community members of seeds in the family *Asteraceae* were all specialists in the phylum Pseudomonadota, while in germinated seeds, the phyla Pseudomonadota and Bacillota were identified as specialists. The endophytic bacterial community members of ungerminated and germinated seeds in the *Solanaceae* and *Brassicaceae* families were specialists in the phylum Bacillota. Finally, in addition to the phylum Pseudomonadota, Cyanobacteriota and Bacillota were also identified as specialist taxa in the endophytic bacterial community of ungerminated and germinated seeds of the *Apiaceae* family, respectively.

### Predicted microbial functions

**Figure 6:**
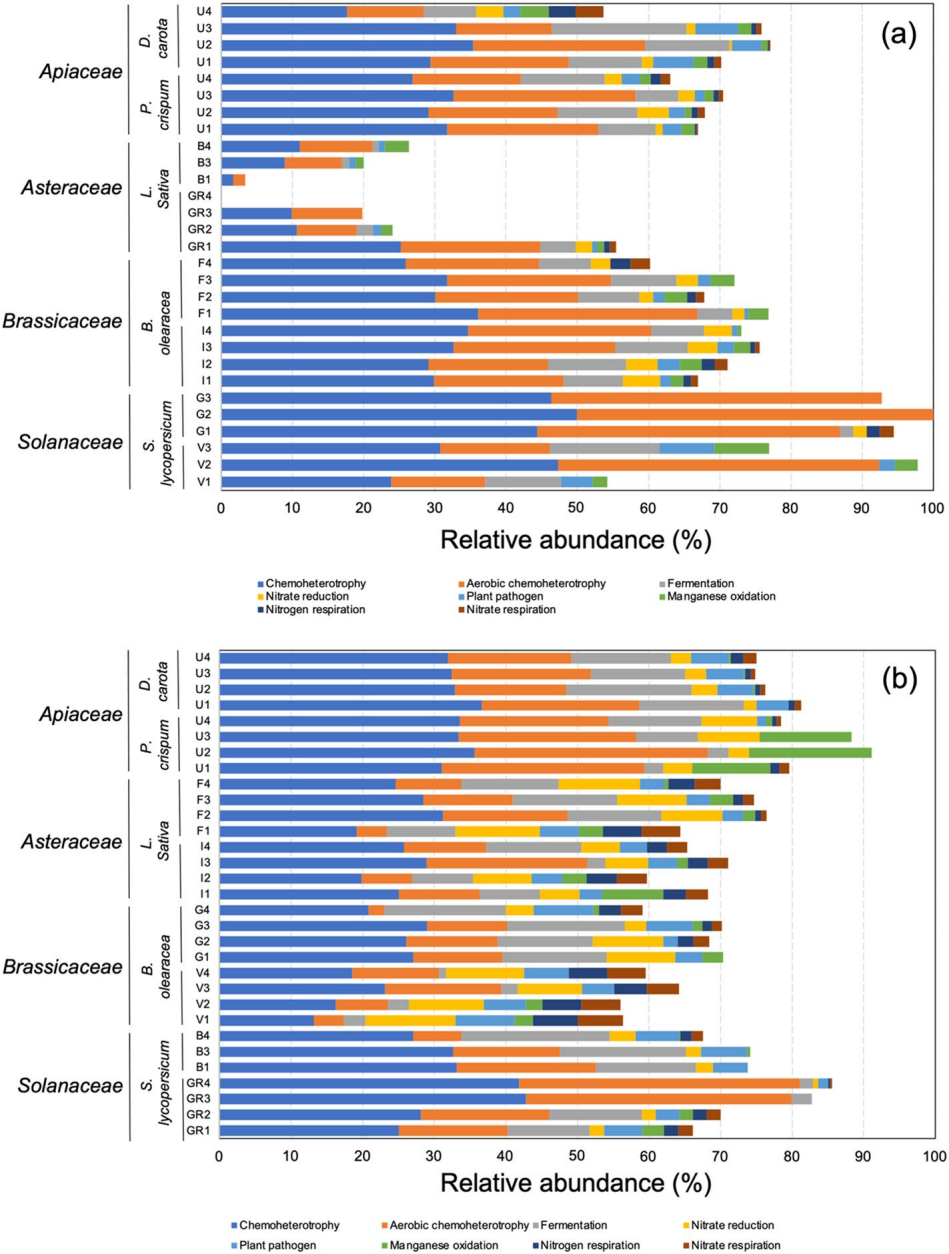
Relative abundances (%) of microbial functional groups of the endophytic bacterial communities in ungerminated seeds (**a**) and germinated seeds (**b**) of the vegetable families: *Apiaceae* (parsley *Petroselinum crispum* and carrot *Daucus carota*, undefined varieties [U]), *Asteraceae* (lettuce, *Lactuca sativa*, ‘Brandelier’ [B] and ‘Grand rapids’ [GR] varieties), *Brassicaceae* (cauliflower and broccoli, *Brassica oleracea*, ‘Fausto’ [F] and ‘Italica’ [I] varieties, respectively), and *Solanaceae* (tomato, *Solanum lycopersicum*, ‘Gladiador’ [G] and ‘Velocity’ [V] varieties).

In relation to predicted microbial functions, in general terms, higher relative abundances were attributed to chemoheterotrophy and aerobic chemoheterotrophy followed by fermentation in both ungerminated and germinated seeds of all studied vegetables, particularly in germinated seeds of members of the *Apiaceae* family, with values ranging from 31 to 37%, 16 to 33% and 3 to 18%, respectively (Fig. 6). Other observed putative functions with higher relative abundances were attributed to nitrate reduction, plant pathogens, manganese oxidation, nitrogen respiration and nitrate respiration.

### PGP traits in isolated endophytic bacteria

**Figure 7:**
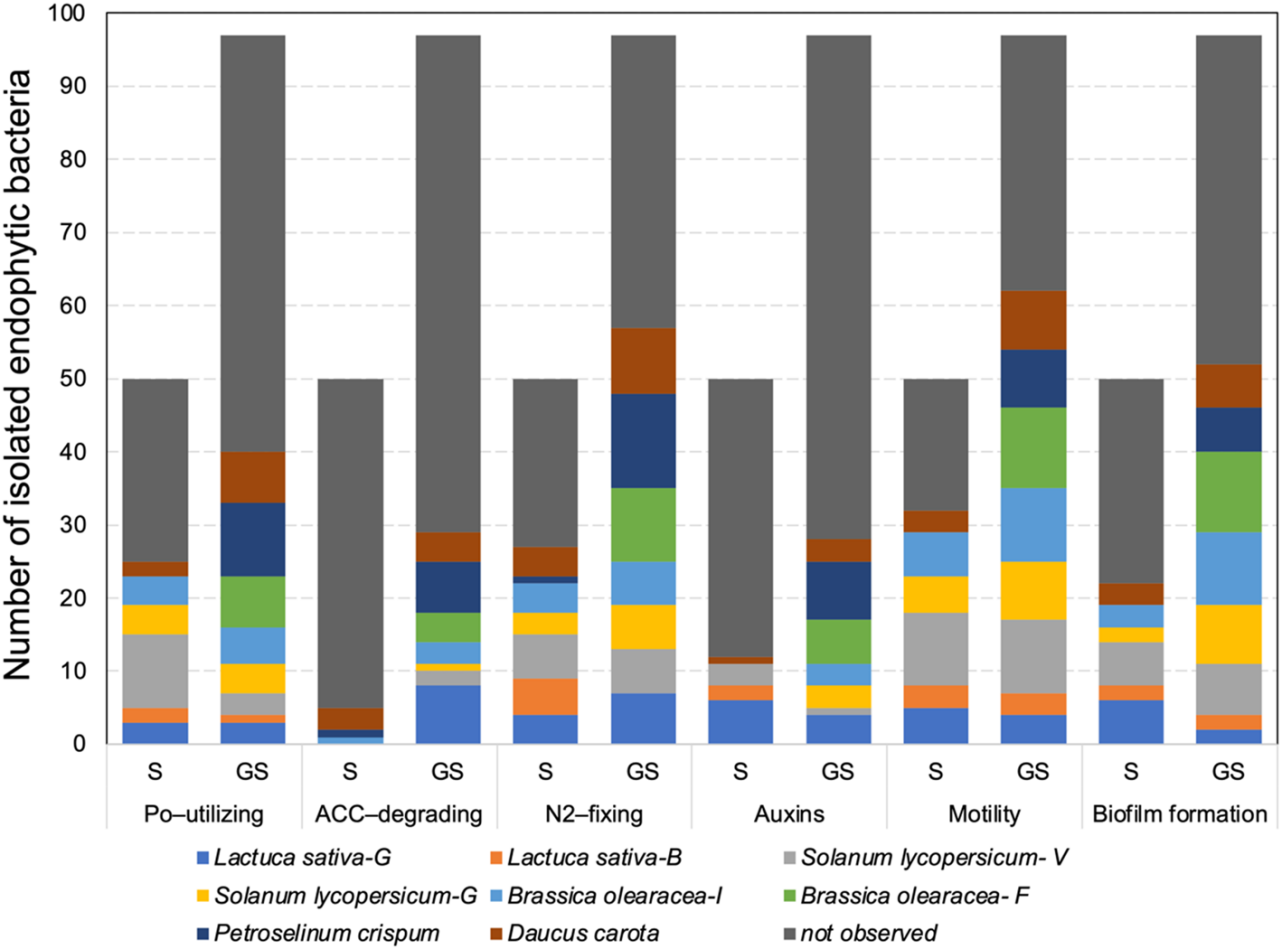
Number of isolated endophytic bacteria from ungerminated seeds (*n* = 50) and germinated seeds (*n* = 97) of each vegetable family showing the plant growth-promoting (PGP) traits. *Po* organic phosphorus, *ACC* 1-aminocyclopropane-1-carboxylate, *N*_*2*_ atmospheric N.

The occurrence of PGP traits in 143 isolated endophytic bacteria (50 from ungerminated seeds and 97 from germinated seeds) was observed in all studied vegetables (Fig. 7). The PGP traits were found in isolates from both ungerminated and germinated seeds with higher abundances for motility (62 strains, equivalent to 64%) and potential N_2_ fixation (57 strains, equivalent to 59%) of germinated seeds and lower relative abundances for 1-aminocyclopropane-1-carboxylate (ACC) deaminase activity (5 strains, equivalent to 10%) and auxin production (12 strains, equivalent to 24%) of ungerminated seeds. Po-utilizing activity varied from 50% in ungerminated seeds to 41% in germinated seeds of isolated endophytic strains. In general terms, endophytic bacteria showing PGP traits were found in both ungerminated and germinated seeds from *L. sativa* (G variety), *B. oleracea* (I variety), *S. lycopersicum* (G variety) and *S. lycopersicum* (V variety, except for ACC deaminase activity). Interestingly, a higher number of endophytic bacteria showing PGP traits were found in germinated seeds of *D. carota*, *P. crispum* and *B. oleracea* (F variety) than in ungerminated seeds.

### Biocontrol activity of isolated endophytic bacteria

**Figure 8:**
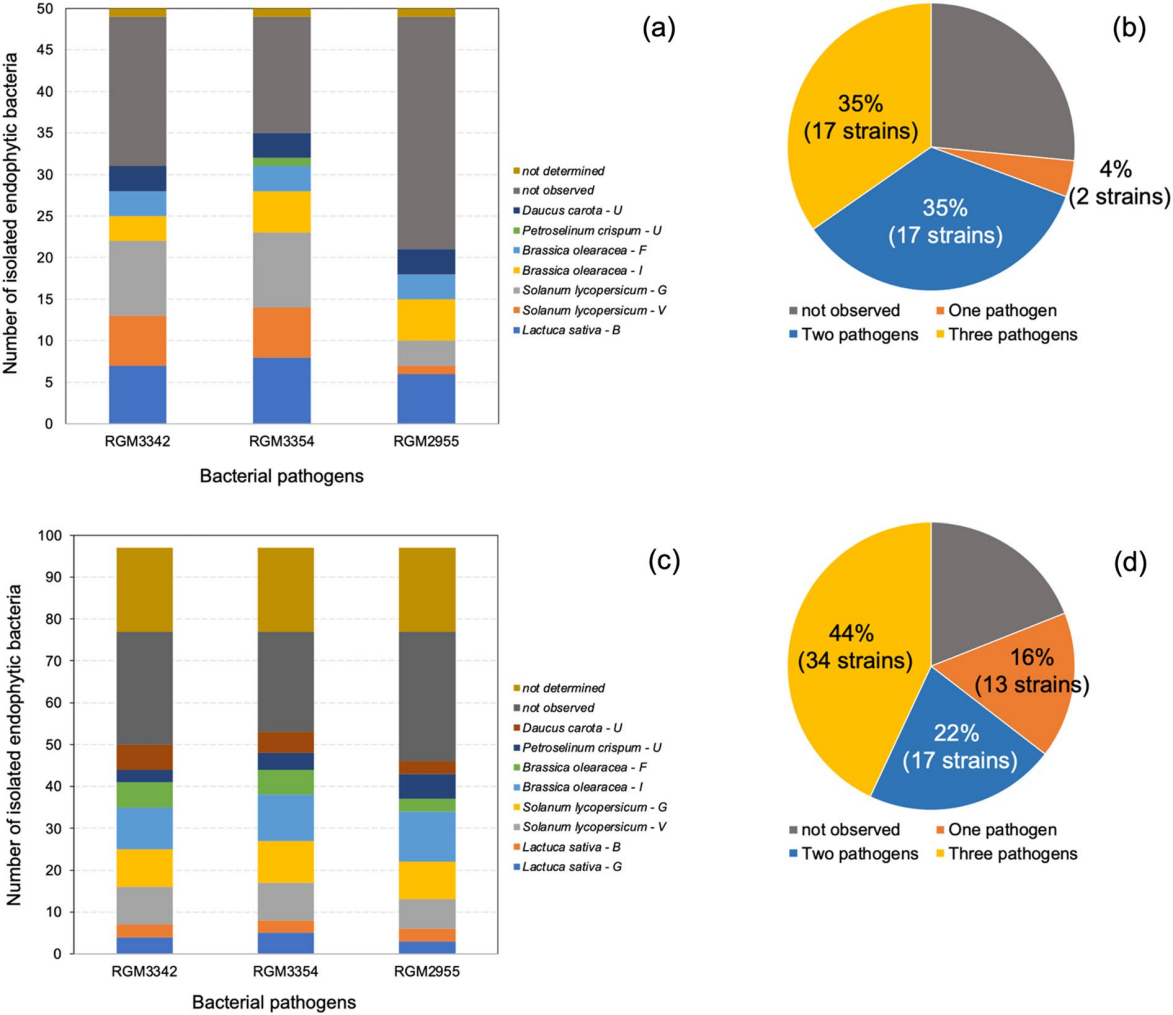
Number of isolated endophytic bacteria from seeds (**a**; *n* = 50) and germinated seeds (**c**; *n* = 97) of each vegetable family showing biocontrol activity on bacterial pathogens of plants. Percentage (%) of endophytic bacterial strains isolated from seeds (**b**) and germinated seeds (**d**) showing single, double, and triple biocontrol activity on bacterial pathogens of plants. RGM3342: *Pseudomonas viridiflava*; RGM3354: *Pseudomonas syringae* pv. *syringae*; RGM2955: *Xanthomonas* sp.

Isolated bacterial strains from ungerminated seeds of all studied vegetables showed biocontrol activity against the three assayed pathogens, except those strains isolated from *P. crispum,* which showed biocontrol activity only against *P. syringae* pv. *syringae* RGM 3354 (Fig. 8a). In general terms, a lower number of isolated bacterial strains showed biocontrol against *Xanthomonas* sp. RGM 2955 (21 strains) compared with *P. syringae* pv. *syringae* RGM 3354 (35 strains) and *P. viridiflava* RGM 3342 (31 strains). In the tested *Pseudomonas*, isolated bacterial strains showing biocontrol activity were mainly isolated from *S. lycopersicum* (both G and V varieties) and *L. sativa* (B variety). Interestingly, 70% of the endophytic bacterial strains from ungerminated seeds showed biocontrol against more than one pathogen assayed (Fig. 8b). Additionally, isolated bacterial strains from germinated seeds of all vegetables also showed biocontrol against the three assayed pathogens (Fig. 8c). Among the three pathogens, isolated bacterial strains showing biocontrol activity were mainly isolated from ungerminated seeds of *S. lycopersicum* (both G and V varieties) and *B. oleracea* (I variety). Similarly, 66% of the endophytic bacterial strains from germinated seeds showed biocontrol against more than one pathogen assayed (Fig. 8d).

### Taxonomic affiliation of selected endophytic bacteria

The taxonomic affiliation of endophytic strains with a great number of PGP traits and biocontrol activity is shown in Table 1. In ungerminated seeds, most isolated strains were affiliated with the genus *Bacillus* (Bacillota), showing organic phosphorus (Po) utilization, N_2_ fixation, and biofilm formation and mainly inhibiting the growth of *P. viridiflava* RGM 3342 and *P. syringae* pv. *syringae* RGM 3354. Other isolated strains with a greater number of PGP traits and biocontrol activity were affiliated with the genera *Rhodococcus* (Actinomycetota phylum), *Paenibacillus* (Firmicota phylum) and *Stenotrophomonas* (Pseudomonadota phylum). In contrast, fewer isolates showed ACC deaminase activity and inhibited *Xanthomonas* sp. RGM 2955. A higher taxonomic diversity of isolates was observed in germinated seeds, where most isolated endophytic strains were affiliated with the *Microbacterium* genus (Actinomycetota phylum), followed by *Bacillus,* showing positive activity in most assayed PGP traits, including ACC deaminase activity. Isolated strains affiliated with the phyla Actinomycetota (*Rhodococcus Arthrobacter*, *Pseudoclavibacter* and *Curtobacterium* genera) and Pseudomonadota (*Pantoea*, *Brucella* and *Stenotrophomonas* genera) were also found to have a greater number of PGP traits and biocontrol activity. Interestingly, members taxonomically close to *Bacillus velezensis* strain FZB42, a recognized gram-positive model strain for plant growth promotion and biocontrol, were isolated in both ungerminated and germinated seeds in our study.

**Table 1:** Taxonomic affiliation of cultured endophytic strains randomly isolated from ungerminated and germinated seeds showing a higher number of plant growth–promoting (PGP) traits and biocontrol activity against bacterial pathogens of plants.

Isolate	Isolation source (family/species/variety)	PGP traits						Biocontrol			Closest relative in the GenBank database (accession no.)*	Similarity (%)	GenBank (accession no.)
		Po–utilizing	ACC–degrading	N2–fixing	Auxins production	Motility	Biofilm formation	RGM 2955	RGM 3354	RGM 3342			
Seeds													
S-30	*Solanaceae*/*Solanum lycopersicum*/velocity	+	–	+	+	+	+	–	+	+	Plant growth-promoting *Bacillus velezensis* strain FZB42 (NR_075005)	99.8	OQ259957
S-1	*Asteraceae*/*Lactuca sativa*/Grand rapids	+	–	+	+	–	+	–	+	+	Carbendazim-degrading *Rhodococcus qingshengii* strain djl-6 isolated from soil (NR_043535)	100	OQ259958
S-14	*Asteraceae*/*Lactuca sativa*/Brandelier	–	–	+	+	+	+	–	+	+	*Paenibacillus solani* strain FJAT-22460 isolated from potato rhizosphere soil (NR_152686)	96.8	OQ259959
S-23	*Solanaceae*/*Solanum lycopersicu*m/Velocity	+	–	–	+	+	+	+	+	+	Plant growth-promoting *Bacillus velezensis* strain FZB42 (NR_075005)	99.9	OQ259960
S-26	*Solanaceae*/*Solanum lycopersicu*m/Velocity	+	–	+	–	+	+	–	+	+	Plant growth-promoting *Bacillus velezensis* strain FZB42 (NR_075005)	100	OQ259982
S-27	*Solanaceae*/*Solanum lycopersicu*m/Velocity	+	–	+	+	–	+	–	+	+	Plant growth-promoting *Bacillus velezensis* strain FZB42 (NR_075005)	100	OQ259983
S-31	*Solanaceae*/*Solanum lycopersicu*m/Velocity	+	–	+	–	+	+	+	+	+	Plant growth-promoting *Bacillus velezensis* strain FZB42 (NR_075005)	99.8	OQ259961
S-34	*Solanaceae*/*Solanum lycopersicu*m/Gladiador	+	–	+	–	+	+	+	+	+	Gamma-polyglutamic acid-producing *Bacillus spizizenii* strain NBRC 101239 isolated from fermented bean (NR_112686)	99.8	OQ259984
S-37	*Brassicaceae*/*Brassica olearacea/*Italica	+	–	+	–	+	+	+	+	+	Gamma-polyglutamic acid-producing *Bacillus spizizenii* strain NBRC 101239 isolated from fermented bean (NR_112686)	99.8	OQ259962
S-38	*Brassicaceae*/*Brassica olearacea/*Italica	+	–	+	–	+	+	+	+	+	Plant growth-promoting *Bacillus velezensis* strain FZB42 (NR_075005)	99.5	OQ259963
S-41	*Brassicaceae*/*Brassica olearacea/*Italica	+	+	+	–	–	+	–	–	+	*Bacillus velezensis* strain GN0015 isolated from soil (OP903018)	98.8	OQ259964
S-48	*Apiaceae*/*Daucus carota/* Undefined	–	+	+	–	+	+	+	+	+	EDTA-degrading *Stenotrophomonas chelatiphaga* strain LPM-5 from sludge (NR_116366)	97.3	OQ259965
S-49	*Apiaceae*/*Daucus carota/* Undefined	+	+	+	–	+	–	+	+	+	Plant growth-promoting *Bacillus velezensis* strain CBMB205 isolated from rice rhizosphere soil (NR_116240)	96.8	OQ259966
Germinated seeds													
P-77	*Apiaceae*/*Petroselinum crispum*/Undefined	+	+	+	+	+	+	+	–	+	Xylanolytic *Microbacterium maritypicum* strain DSM 12,512 isolated from forest soil (NR_042351)	99.6	OQ259967
P-36	*Brassicaceae*/*Brassica olearacea*/Italica	+	+	+	–	+	+	+	+	+	Carbendazim-degrading *Rhodococcus qingshengii* strain djl-6 isolated from soil (NR_043535)	99.7	OQ259985
P-37	*Brassicaceae*/*Brassica olearacea*/Italica	+	–	+	+	+	+	+	+	+	Plant growth-promoting *Bacillus velezensis* strain FZB42 (NR_075005)	98.4	OQ259986
P-56	*Brassicaceae*/*Brassica olearacea*/Fausto	+	+	+	+	–	+	+	+	+	*Arthrobacter bambusae* strain THG-GM18 isolated from bamboo soil (NR_133968)	98.1	OQ259968
P-61	*Brassicaceae*/*Brassica olearacea*/Fausto	+	+	+	–	+	+	–	+	+	Carbendazim-degrading *Rhodococcus qingshengii* strain djl–6 isolated from sludge (NR_115708)	99.9	OQ259969
P-67	*Apiaceae*/*Petroselinum crispum/*Undefined	+	–	+	+	+	+	+	–	–	*Pantoea agglomerans* strain ATCC 27155 (NR_114505)	99.2	OQ259987
P-75	*Apiaceae*/*Petroselinum crispum/*Undefined	+	+	+	+	+	+	–	–	–	*Pantoea vagans* strain LMG 24199 isolated from Eucalyptus (NR_116115)	98.9	OQ259988
P-78	*Apiaceae*/*Petroselinum crispum/*Undefined	+	+	+	–	+	+	+	+	+	Bacterium strain BS1008 isolated from tomato rhizosphere (MK824196)	98.7	OQ259970
P-81	*Apiaceae*/*Petroselinum crispum/*Undefined	+	–	+	+	+	+	+	+	+	*Paenibacillus nicotianae* strain YIM h-19 isolated from a tobacco (NR_134783)	98.4	OQ259971
P-88	*Apiaceae*/*Daucus carota*/Undefined	+	+	+	+	+	+	–	–	–	*Microbacterium foliorum* strain P 333/02 isolated from grass phyllosphere (NR_025368)	99.6	OQ259972
P-90	*Apiaceae*/*Daucus carota*/Undefined	+	+	+	+	–	+	–	+	–	*Pseudoclavibacter terrae* strain THG-MD12 isolated from dwarf lilyturf rhizosphere soil (NR_145621)	98.6	OQ259989
P-94	*Apiaceae*/*Daucus carota*/Undefined	+	+	+	–	+	+	+	+	+	*Microbacterium foliorum* strain P 333/02 isolated from grass phyllosphere (NR_025368)	99.6	OQ259990
P-13	*Asteraceae*/*Lactuca sativa*/Grand rapids	–	+	+	+	+	–	–	+	–	*Curtobacterium herbarum* strain P 420/07 isolated from grass phyllosphere (NR_025461)	99.9	OQ259991
P-19	*Solanaceae*/*Solanum lycopersicum*/Velocity	+	+	+	–	+	–	+	+	–	*Brucella grignonensis* strain OgA9a isolated from soil (NR_028901)	99.4	OQ259992
P-22	*Solanaceae*/*Solanum lycopersicum*/Velocity	–	+	+	–	+	+	+	+	+	*Microbacterium oxydans* strain DSM 20578 isolated from air (NR_044931)	99.9	OQ259973
P-23	*Solanaceae*/*Solanum lycopersicum*/Velocity	+	–	–	+	+	+	–	+	+	Plant growth-promoting *Bacillus velezensis* strain FZB42 (NR_075005)	95.1	OQ259974
P-28	*Solanaceae*/*Solanum lycopersicum*/Velocity	+	–	+	–	+	+	+	+	+	Plant growth-promoting *Bacillus velezensis* strain FZB42 (NR_075005)	99.8	OQ259975
P-29	*Solanaceae*/*Solanum lycopersicum*/Gladiador	+	–	+	+	+	+	–	–	–	Plant growth-promoting *Bacillus velezensis* strain FZB42 (NR_075005)	100	OQ259993
P-39	*Brassicaceae*/*Brassica olearacea*/Italica	+	–	–	+	+	+	+	+	+	*Stenotrophomonas panacihumi* strain MK06 isolated from ginseng soil (NR_117406)	98.6	OQ259976
P-48	*Brassicaceae*/*Brassica olearacea*/Italica	–	–	+	+	+	+	+	+	+	Plant growth-promoting *Bacillus velezensis* strain FZB42 (NR_075005)	98.3	OQ259994
P-57	*Brassicaceae*/*Brassica olearacea*/Fausto	+	–	+	+	+	+	+	+	+	Xylanolytic *Microbacterium maritypicum* strain DSM 12512 isolated from forest soil (NR_042351)	99.8	OQ259977
P-71	*Apiaceae*/*Petroselinum crispum*/Undefined	+	+	+	+	–	–	+	+	–	Endophytic Microbacterium zeae strain 1204 isolated from maize stem (NR_149816)	97.7	OQ259978
P-76	*Apiaceae*/*Petroselinum crispum*/Undefined	+	+	+	–	+	+	–	–	–	*Pantoea vagans* strain LMG 24199 isolated from Eucalyptus (NR_116115)	98.3	OQ259979
P-93	Apiaceae/Daucus carota/undefined	+	–	+	–	+	+	+	+	+	*Microbacterium foliorum* strain P 333/02 isolated from grass phyllosphere (NR_025368)	99.3	OQ259980
P-96	Apiaceae/Daucus carota/undefined	+	–	+	–	+	+	–	–	+	*Stenotrophomonas maltophilia* strain ATCC 13637 isolated from soil (NR_112030)	98.5	OQ259981

*Po* organic phosphorus, *ACC* 1-aminocyclopropane-1-carboxylate, *N*_*2*_ atmospheric nitrogen, *RGM 2955* *Xanthomonas* sp. RGM 2955, *RGM 3354* *Pseudomonas syringae* pv. *syringae* RGM 3354, *RGM 3342* *Pseudomonas viridiflava* RGM 3342. + positive, – negative.

*Based on partial sequencing of 16S rRNA gene and comparison with those present in GenBank database from National Center for Biotechnology Information (NCBI) by using BLAST nucleotide tool.

## Discussion

The copy numbers of 16S rRNA genes g^–1^ sample by qPCR suggest a wide range of bacterial abundance (10^4^ to 10^8^) inside the studied ungerminated and germinated seeds. Significant variations in 16S rRNA genes per g^–1^ seed (10^6^ to 10^8^) have been observed in different species of herbs, vines and trees by using qPCR^23^. In members of *Brassicaceae*, our counts were lower than those observed in *Brassica napus* L., *Brassica juncea* (L.) Czern. and *Brassica rapa* L. (10^6^ to 10^7^ 16S rRNA gene copies g^–1^ seed)^24^. Similarly, Wassermann et al.^25^ observed higher bacterial densities (10^8^ to 10^11^ 16S rRNA gene copies number) in seeds of *Brassica napus*. Although relatively low densities of bacteria in seeds (10^2^ to 10^3^ CFU g^–1^) have been reported^26^, the lower bacterial counts could also be modulated by the disinfecting and packing treatments that seeds are subjected to. In this context, disinfection significantly reduced the bacterial population sizes (10^4^ to 10^8^ bacterial cells g^–1^) to undetected levels in pregerminated seeds and 5-day emerged roots of maize^27^.

Regarding the endophytic bacterial community, alpha diversity (Chao1, Shannon, and Simpson indices) did not show significant differences between ungerminated and germinated seeds, except in members of *Solanaceae*. Similar to our observations, significant differences between seeds and seedlings were not observed in rice^28^. In particular, the Shannon index values in our study (3 to 8) were higher than those found in seeds of different species of barley (2 to 4)^29^, rice (1 to 3)^28^, and chickpea (1 to 3)^30^. Higher values of the Chao1 index were also obtained (10 to 250) compared with those values (< 20) observed in seeds of bean and radish^31^. A study in tomato seeds revealed higher values for Chao1 (2500 to 7500) but similar Shannon values (4 to 7) than those observed in our study^32^. The significantly lower values of diversity in germinated seeds than ungerminated seeds in *Solanaceae* suggest that the diversity of taxa could tend to decrease after germination in this family.

Our results also showed Pseudomonadota, Bacillota and Actinomycetota as the dominant taxa in both ungerminated and germinated seeds. The same taxa have commonly been reported as a dominant phylum in the microbiome of ungerminated and germinated seeds of diverse plants, including members of the four studied vegetables^33–36^. Members of the phyla Bacteroidota, Cyanobacteriota and Planctomycetota were observed in the studied samples at lower relative abundances. Bacteroidota and Planctomycetota have been reported in seeds and emerged seedlings of plants gnotobiotically grown^37,38^. Similarly, studies have described the influence of cyanobacteria on the seed vigor of maize and seedling emergence of native *Fabaceae* and *Poaceae* plants^39,40^.

The role of the microbiome in seeds and germination has been widely discussed, supporting the relevance of seed-borne bacteria for fitness, survival, and stress tolerance in the early stages of plants^9,41,42^. In this context, chemoheterotrophy and fermentation were the dominant functions predicted in both ungerminated and germinated seeds. Functions involved in nitrogen cycling (nitrate reduction and respiration of nitrogen and nitrate) were also predicted but at lower relative abundances. Most bacterial populations associated with plants are chemoheterotrophs, particularly in seeds and fruits^43^. Similarly, fermentative bacteria, such as lactic acid bacteria, are ubiquitous in seeds and sprouts with the potential to improve seed germination and prevent the attack of pathogens^44,45^. In addition, bacteria showing nitrate reduction functions have also been observed and isolated from rice seeds^38^. Our results showed similar functional profiles between ungerminated and germinated seeds, which could be predicted using FAPROTAX. This result should be taken with caution because it has been proposed that the database size and low taxonomic identification are the main factors that limit FAPROTAX application^46^. Therefore, a gap may exist between the microbial functional profile predicted by FAPROTAX and the real ecological role of bacterial communities in plant and seed microbiomes.

Finally, the SPEC–OCCU plot identified Pseudomonadota and Bacillota as specialist taxa in ungerminated and germinated seeds. Members of the phyla Pseudomonadota and Bacillota have commonly been reported as part of the microbiome of seeds, and some studies have proposed them as keystone taxa, which can be transmitted to seedlings, in seeds of the *Solanaceae*, *Brassicaceae* and other plant families^10,35,47,48^.

Our study revealed microbiome dynamics associated with the germination of commercial seeds and probably linked to the physiological changes in the studied vegetables. Studies have showed temporal dynamics in the structure, relative abundance, and alpha diversity of seed-borne bacterial communities in early plant growth stages^27,30,31,49^. Our results are also in agreement with those differences observed in the beta diversity of endophytic bacterial communities between seeds and early plant growth stages, seedlings, and maturation, including some varieties or cultivars of *Solanaceae* and *Brassicaceae*^7,32,35^.

During the last decade, the plant microbiome has gained substantial interest due to the development of sustainable, microbe-based alternatives to chemical pesticides and fertilizers for crop production. Furthermore, beneficial plant microorganisms help to reduce plant stress under a climate change scenario^50,51^. The presence of PGP traits in endophytic bacteria isolated from ungerminated and germinated seeds was also observed in our study. As discussed above, seeds and spermospheres contain numerous beneficial bacteria that provide nutrients and biomolecules pivotal for the germination, plant growth and suppression of seed-borne diseases^17,26,34^. In this context, studies have reported bacteria in seeds harboring PGP functions such as phosphorus solubilization, atmospheric nitrogen fixation, antibiosis against pathogens, acetoin secretion, ACC deaminase activity, production of auxins (e.g., indole acetic acid), and production of siderophores^41,45,52^, particularly those with the potential to be transmitted across plant genotypes, habitats, and generations from seeds^38^. In relation to the biotechnological level, an approach was successfully developed by Mitter et al.^19^ for the modification of plant microbiomes and growth traits of plants by introducing selected beneficial bacteria (endophytic *P. phytofirmans* PsJN) at flowering into the progeny seed microbiome. This advance provides new opportunities in crop breeding by the selection and delivery of specific endophytic bacteria into plants to overcome limitations in agricultural production.

We observed that most of the isolated strains were affiliated with the genus *Bacillus* in seeds, particularly *Bacillus velezensis* strain FZB42 (accession no. NR_075005). Thus, *Bacillus* have been widely recognized as a valuable tool for agriculture^53,54^, where *Bacillus velezensis* strain FZB42 is studied as a model of PGP bacteria and biocontrol agent against plant pathogens^55^. In germinated seeds, the most isolated strains were affiliated with *Microbacterium*. *Microbacterium* species have also been described as efficient PGP bacteria under biotic and abiotic stresses^56,57^. The remaining strains show affiliation with the genera *Rhodococcus*, *Arthrobacter*, *Pseudoclavibacter*, *Curtobacterium*, *Pantoea*, *Brucella* and *Stenotrophomonas*, most of which are known to comprise endophytes or species/strains relevant for crop improvement^52,58,59^.

With respect to the composition of endophytic bacterial communities obtained in ungerminated and germinated seeds by cultivation, we observed that most endophytic isolates belonged to the phylum Bacillota (*Bacillus*) in seeds, while members of the phyla Actinomycetota (*Rhodococcus*, *Microbacterium*, *Arthrobacter Pseudoclavibacter*, *Curtobacterium*) and Pseudomonadota (*Pantoea*, *Brucella* and *Stenotrophomonas*) were mostly isolated from germinated seeds. In contrast, DNA metabarcoding analysis showed that Pseudomonadota and Bacillota were dominant taxa in both ungerminated and germinated seeds. This result not only revealed the differences between both methods used but also indicated the differences regarding the cultivability of isolated bacterial strains between ungerminated and germinated seeds under our experimental conditions.

Interestingly, a higher number of PGP traits was observed in strains isolated from germinated seeds than in those from ungerminated seeds. In addition, a high percentage of the isolated strains also showed biocontrol activities against certified bacterial pathogens in Chile, preventing the growth on agar plates of at least one of three assayed pathogens (*Xanthomonas* sp. RGM 2955, *P. syringae* pv. *syringae* RGM 3354 and *P. viridiflava* RGM 3342). As a result of climate change, plant pathogens and emerging diseases are one of the main concerns and challenges in modern agriculture and food security worldwide^60^. As a countermeasure, crop migration has been adopted by farmers, which has also provoked the migration and higher severity of pathogen attacks and/or the emergence of novel diseases and pests affecting crop yields. Chile is not an exception, and therefore, the production of commercial seeds harboring beneficial microbiomes, including local or emerging pathogen–controlling bacteria, has become an attractive strategy to be adopted under ‘One Health’ and ‘Food Safety’ approaches^61^. Both approaches are promoted by the World Health Organization (WHO) as a response to the recently increasing concern of emerging zoonotic disease outbreaks (https://www.who.int/europe/initiatives/one-health) and by the Food and Agricultural Organization (FAO) as part of agri-food system transformation to prevent food containing microbes and substances that could harm public health and the environment (https://www.fao.org/food-safety/background/qa-on-food-safety/en/).

## Material and methods

### Seeds and germinated seeds

The commercial seeds used in this study were provided by Chile’s National Association of Seed Producers (ANPROS A.G.; https://www.anproschile.cl/) and purchased from a Chilean seed distributor. The seeds of the following vegetables were studied: *Apiaceae* (parsley *P. crispum* and carrot *D. carota*, U variety), *Asteraceae* (lettuce, *Lactuca sativa*, B and GR varieties), *Brassicaceae* (cabbage and broccoli, *B. oleracea*, F and I varieties, respectively), and *Solanaceae* (tomato, *S. lycopersicum*, G and V varieties). The seeds were produced in the central region of Chile and packed by Bernagroup SPA (parsley and carrot), ANASAC (https://www.anasac.cl/) (lettuce, tomato [V variety] and broccoli) and Bioamerica S.A. (https://www.bioamerica.cl/) (tomato [G variety] and cabbage).

Under clean bench conditions, 40 seeds (0.04 to 0.09 g of parsley or carrot, 0.03 to 0.05 g of lettuce, 0.15 to 0.16 g of cabbage or broccoli, and 0.08 to 0.1 g of tomato) were randomly chosen, weighed, and gently washed with sterile distilled water for 5 min. The seeds were then disinfected by immersion in ethanol (75%) for 2 min followed by immersion in sodium hypochlorite (2.5 vol vol^–1^) for 2 min, repeatedly rinsed with sterile distilled water for 5 min, and used in further analysis^62^. In addition, aliquots of 200 µL of the last rinse were inoculated in glass tubes containing 5 mL of tenfold diluted Luria–Bertani broth (LB; Oxoid Ltd., Hampshire, UK) and incubated at 30 °C for one week to confirm the absence of culturable (viable) seed epiphytic bacteria.

Ten disinfected seeds were taken and germinated per species (set of 10 seeds × 8 vegetable species × quadruplicate = 32 samples) in sterile 150 mL glass bottles containing 50 mL of Murashige & Skoog Basal Medium with Vitamin and Sucrose (Phytotech Labs®, Lenexa, KS, USA)-supplemented agar (15 g L^–1^; Oxoid Ltd.) and amphotericin B (4 µg mL^–1^; Corning, Manassas, VA, USA) to prevent fungal growth. The germination was performed under darkness at room temperature in a Plant Growth Chamber model BJPX-A300 (BIOBASE, Shandong, China). After approximately two weeks of germination, all seeds (10) were germinated (or sprouts) in the cotyledon stage and then used for further analyses*.*

### Sample processing

The remaining disinfected ungerminated seeds (set of 30 seeds × 8 vegetable species × quadruplicate = 32 samples) and germinated seeds were processed as follows. Thirty disinfected ungerminated seeds were aseptically macerated and resuspended in 1 mL of sterile saline solution (SSS; 0.85% NaCl) into sterile 2 mL plastic tubes. In parallel, sets of 10 germinated seeds (in quadruplicate) were aseptically weighed, macerated, and resuspended in 1 mL of SSS. The ungerminated and germinated seed suspensions were vigorously homogenized for 5 min and then decanted for 5 min until a solid (lower) and liquid (upper) phase was obtained. The solid phases were used to extract DNA for molecular methods, while liquid phases were recovered and transferred to new tubes, and aliquots were spread onto agar plates to isolate endophytic bacteria by cultivation as described below.

### DNA extraction

The solid phases of the ungerminated and germinated seed suspensions were collected (without a liquid phase), transferred to new sterile 2 mL plastic tubes, and used for DNA extraction with a DNeasy PowerSoil kit according to the manufacturer’s instructions (Qiagen, Inc., Germantown, MD, USA). The selection of this kit was based on the quantity and quality of DNA extracts obtained after preliminary testing with other commercial kits and our seeds and plant tissues (E.Z.N.A.® SP Plant DNA Kit, Omega Biotek, Inc., Nocross, GA, USA; Quick-DNA™ Plant/Seed Miniprep Kit, Zymo Research, Irvine, CA, USA). The quantity and quality of DNA in extracts were estimated with a Qubit 4 fluorometer and Invitrogen Qubit assay kit (Thermo Fisher Scientific, Waltham, VT, USA), and only extracts exhibiting an A260/A280 absorbance ratio of ~ 1.8, as revealed by a Multiskan™ GO microplate spectrophotometer (Thermo Fisher Scientific, Inc.), were selected for the following molecular analysis. DNA was extracted once from each recovered solid phase per sample.

### Abundance of endophytic bacteria

The 16S rRNA was used as a gene target to estimate the abundance of bacteria in ungerminated and germinated seeds by quantitative PCR (qPCR) by using a universal primer set for the bacterial 16S rRNA gene, Bac1369F (5′-CGG TGA ATA CGT TCY CGG-3′) and Prok1492R (5′-GGW TAC CTT GTT ACG ACT-3′) as previously described by Zhang et al.^63^. The 16S rRNA genes were amplified with a StepOne Real-Time PCR System (Thermo Fisher Scientific, Inc.) and PowerUpTM SYBR™ Green Master Mix (Applied BiosystemsTM, Foster City, CA, USA) using ~ 25 ng of DNA μL^−1^. The copy number of 16S rRNA genes was calculated using *Escherichia coli* standards built with dsDNA gBlock® Gene Fragments (Integrated DNA Technologies, Inc. Iowa, USA) and the equation ([concentration of the dsDNA gBlock® Gene Fragment in ng μL^−1^] × [molecular weight in fmol ng^−1^] × [Avogadro's number] = copy number) described by Whelan et al.^64^. Based on the standard curves, absolute quantification (AQ) of 16S rRNA genes was expressed as gene copy number per gram of fresh weight of seed or tissue (gene copy g^−1^ seed or tissue). The data obtained from quantification were nonnormally distributed; thus, qPCR values were contrasted by the Kruskal–Wallis test.

### Endophytic bacterial community

The DNA metabarcoding analysis was performed using Illumina’s platform as described by Yarimizu et al.^65^. Aliquots of 2.5 µL of each DNA extract were used as templates, and the V3 ~ V4 region of the 16S rRNA gene was amplified with the Bakt_341f (5′-CCT ACG GGN GGC WGC AGA CAC TCT TTC CCT ACA CGA CGC TCT TCC GAT CT-3′) and Bakt_805r (5′-GAC TAC HVG GGA CTG GAG TTC AGA CGT GCT CTT CCG ATC T-3′) primer sets using Takara MightyAMP™ Hotstart DNA Polymerase (Takara Bio, Inc., Shiga, Japan). PCR products were purified using a Pronex® Size Selective Purification System and indexed with KAPA HiFi HotStart ReadyMix (F. Hoffmann-La Roche Ltd., Basel, Switzerland) and a Nextera v2 kit (Illumina Inc., USA). Then, the lengths of indexed DNA fragments were verified with an Agilent TapeStation 4150 fragment analyzer (Agilent, Inc., Santa Clara, CA, USA) using a D1000 Screentape kit and pooled and mixed with 20% PhiX Sequencing Control v3 as an internal control. Finally, the indexed 16S rRNA gene libraries were loaded into MiSeq Kit V3 (600-cycles) and sequenced with a MiSeq System (Illumina, Inc., San Diego, CA, USA) provided by the Scientific and Technological Bioresources Nucleus at Universidad de La Frontera (BIOREN-UFRO; https://bioren.ufro.cl/).

The sequencing data were subjected to bioinformatic and statistical analyses as follows. First, the raw sequence reads were trimmed and processed using SHI7 software to obtain high-quality data^66^, and then Silva123 was used to align the processed sequences by NINJA-OPS^67^. Chloroplast and mitochondrial DNA sequences were removed using QIIME1, and filtered data were further standardized by normalizing with cumulative sum scaling (CSS)^68^. The relative abundance of microbial species in each sample was calculated and represented by a stacked figure. Bacterial diversity was assessed using alpha-diversity indices (e.g., Chao1, Shannon and Simpson), which were calculated using QIIME1. Then, the significant differences between ungerminated and germinated seeds of the same vegetable family were analyzed by the Kruskal–Wallis test. Principal coordinates analysis (PCoA) based on the Bray–Curtis distance was used to ordinate the samples by the R project ‘*vegan*’ package^11^, and differences were evaluated by Adonis (*p* < 0.05). Moreover, the specificity and occupancy of community members were calculated in R, and specialist taxa were identified based on these indicators^69,70^. In addition, to predict the function of the microbial community, we used the FAPROTAX database and software (https://pages.uoregon.edu/slouca/LoucaLab/archive/FAPROTAX/lib/php/index.php), which estimates putative metabolic or other ecologically relevant functions based on the current literature on cultured strains as described by Louca et al.^71^. Data were visualized with the ‘*ggplot2*’ package in R 4.2.1 (https://www.r-project.org/). The R codes used during data analysis are presented in the Supplementary Material.

### Isolation of endophytic bacteria

The liquid phases of the obtained ungerminated and germinated seed suspensions were serially diluted (10^−1^ to 10^−4^) in SSS and plated (in quadruplicate) onto Petri dishes containing tenfold diluted LB, minimal medium NM1 and R2A medium (Oxoid Ltd.) supplemented with agar (15 g L^–1^) and amphotericin B (4 µg mL^–1^; Sigma‒Aldrich). The agar plates were incubated for one week at 30 °C, and then a total of 50 colonies from S and 97 colonies from germinated seeds were randomly chosen based on colony phenotype (size, whole shape, edge, color, elevation, etc.), purified by streaking on agar plates, coded and stored at − 80 °C in glycerol:LB medium (3:7).

### Screening of PGP traits in isolated endophytic bacteria

Traditional culture methods were used for screening plant growth-promoting (PGP) traits in isolated endophytic strains. The utilization of insoluble Po was assayed on agar plates containing phytase-screening medium (10 g L^−1^ d-glucose, 4 g L^−1^ sodium phytate, 4 g L^−1^ CaCl_2_, 5 g L^−1^ NH_4_NO_3_, 0.5 g L^−1^ KCl, 0.5 g L^−1^ MgSO_4_ × 7H_2_O, 0.01 g L^−1^ FeSO_4_ × 7H_2_O, and 0.01 g L^−1^ MnSO_4_ × H_2_O)^72^. The endophytic strains were grown for a week at 30 °C, and clear zones surrounding colonies were considered positive for Po − activity. ACC deaminase activity, which is related to the ability of bacteria to reduce stress in plants, was assayed according to Penrose and Glick ^73^. Aliquots (10 µL) of washed cells from fresh culture of each isolate were placed on DF minimal salt agar medium containing 3 mM ACC (Santa Cruz Biotechnology, Inc., Dallas, TX, USA) as the sole nitrogen (N) source and incubated for one week at 30 °C. The growth of endophytic strains on DF medium supplemented with ACC was considered positive for ACC − deaminase activity.

The putative ability to fix atmospheric N (N_2_) among isolated endophytic strains was screened on NFb (nitrogen–free broth) semisolid culture medium, as previously used by Astorga-Eló et al. ^74^. Bacterial growth as a ‘veil-like’ pellicle under the medium surface was considered a putative N_2_ fixing strain. Finally, the production of tryptophan-induced auxins, which is related to plant root growth, was determined by using the Salkowski colorimetric method according to the protocol described by Astorga-Eló et al. ^74^. Aliquots (10 µL) of fresh cultures of each isolate were transferred to LB broth supplemented with 5 mM tryptophan (Sigma‒Aldrich). After incubation (2 weeks at 30 °C under shaking [80 rpm]), bacterial cells were removed by filtration (0.22 µm; mixed cellulose esters [MCE] membrane; Jet Bio-Filtration Co., Ltd., Guangzhou, China), and then 50 µL of filtrates was mixed with 100 µL of Salkowski’s reagent (150 mL of concentrated H_2_SO_4_, 250 mL of distilled H_2_O, 7.5 mL of 0.5 M FeCl_3_ × 6H_2_O) and incubated for 30 min at room temperature under darkness. The content of tryptophan − induced auxins was determined by absorbance at 530 nm with a standard curve, and the presence of red color (≥ 0.1 µg L^−1^ value) was considered a positive result.

In addition to functional PGP traits, vitality and cooperative traits have also been described as desirable characteristics in beneficial bacteria for agricultural sustainability. In this context, motility and biofilm formation in isolated endophytic strains were also screened. Motility was determined in Petri dishes with semi-solid agar media (3 g L^−1^ beef extract, 10 g L^−1^ pancreatic digest of casein, 5 g L^−1^ sodium chloride, 5 mL L^−1^ of 1% triphenyltetrazolium chloride solution, and 400 g L^−1^ agar) as described by Ji et al.^75^. Each isolated endophytic strain was spotted on the center of a semisolid agar media plate using a sterile needle, and then, the plates were incubated at 30 °C for one week. Bacterial motility was assessed by measuring the diameter of the area that bacteria were spread out from the center of the plate. In parallel, biofilm formation was assayed in glass tubes containing 5 mL of fresh LB culture of each isolated endophytic strain and incubated at 30 °C for 1 day under shaking (80 rpm). The presence of a floating film at the air–broth interface after 3 days at 30 °C under static conditions was considered positive according to Aya Castañeda et al.^76^.

### Screening of biocontrol activity in isolated endophytic bacteria

Certified bacterial pathogens of plants were purchased from The Chilean Collection of Microbial Genetic Resources (www.cchrgm.cl) managed by The Agricultural Research Institute of Chile (INIA). The pathogens *Xanthomonas* sp. RGM 2955, *Pseudomonas syringae* pv. *syringae* RGM 3354 and *Pseudomonas viridiflava* RGM 3342^77,78^ were used as models to evaluate the biocontrol activity as follows.

The streak method^79^ was used for a rapid screening of the antimicrobial activity of each isolated endophytic strain against the bacterial pathogens on LB agar plates. The endophytic strain of interest was seeded by streaking in a border of the agar plate and incubated for 1 day at 30 °C. After incubation, the agar plate was seeded with the three pathogens tested by a single streak perpendicular to the streak of the strain of interest. After further incubation depending upon the strain (1 to 2 days), the antimicrobial interactions were analyzed, and those endophytic strains showing evident inhibition zones on agar plates were considered positive for biocontrol activity against the tested pathogens. In addition, those endophytic strains showing fast and invasive growth (e.g., swarming) and covering the pathogens a few hours after their inoculation were considered ‘not determined’.

### Taxonomic affiliation of selected endophytic bacteria

Thirty–eight endophytic strains showing a high number of assayed PGP traits and biocontrol activity were selected and taxonomically classified based on partial sequencing of 16S rRNA genes as described by Acuña et al.^80^.

Chromosomal DNA from fresh cultures of each isolate was extracted using a DNeasy® PowerSoil® Pro Kit (Qiagen, Inc., Germantown, MD, USA), and the 16S rRNA genes were amplified by PCR using the primers 27f (5′-AGA GTT TGA TCC TGG CTC AG-3′) and 1492r (5′-TAC GGY TAC CTT GTT ACG ACT T-3′)^81^. PCR conditions included an enzyme activation step at 95 °C for 2 min, followed by 35 cycles of 30 s of denaturation at 95 °C, 1 min of annealing at 55 °C, 2 min of extension at 72 °C, and a final extension for 10 min at 72 °C. PCRs were performed with a MultiGene™ OptiMax thermal cycler (Labnet International, Inc., Edison, NJ, USA) using GoTaq® G2 Feli DNA polymerase (Promega, Madison, WI, USA). Finally, PCR products were sent to Macrogen, Inc. (Seoul, Korea) for purification and sequencing in both directions, upstream (toward the 5′ end) or downstream (toward the 3′ end). Then, sequences were trimmed and filtered using Geneious Prime software (Auckland, New Zealand), and consensus sequences (> 800 bp) were used for taxonomic affiliation at the genus level (> 90% identity) using the 16S rRNA gene microbial reference database of the GenBank database (https://www.ncbi.nlm.nih.gov/genbank/) from The NCBI.

### Ethics approval

All sampling procedures and methods involved in this study were revised and approved with certification No. 017_20 (issued on April 1st, 2020) by The Scientific Ethics Committee from Universidad de La Frontera (CEC-UFRO; https://cec.ufro.cl/), which is certified by the Health Ministry of the Chilean Government (MINSAL; https://www.minsal.cl/), according to relevant Chilean and international guidelines and regulations.

### Supplementary Information

Supplementary Information.

## Data Availability

Raw sequencing data from DNA metabarcoding analysis were deposited in the Sequence Read Archive (SRA; https://www.ncbi.nlm.nih.gov/sra) from the National Center for Biotechnology Information (NCBI) under accession number PRJNA926007. The 16S rRNA gene sequences from the selected endophytic bacterial strains were deposited in GenBank (https://www.ncbi.nlm.nih.gov/genbank/) under accession numbers OQ259957 to OQ259994.
